# Improving programme‐led and focused interventions for eating disorders: An experts' consensus statement—A UK perspective

**DOI:** 10.1002/erv.2981

**Published:** 2023-05-22

**Authors:** Emily Davey, Karina Allen, Sophie D. Bennett, Rachel Bryant‐Waugh, Tim Clarke, Zafra Cooper, Katharina Dixon‐Ward, Jake Dudley, Ivan Eisler, Jess Griffiths, Andrew J. Hill, Nadia Micali, Rebecca Murphy, Ivana Picek, Ros Rea, Ulrike Schmidt, Mima Simic, Kate Tchanturia, Gemma Traviss‐Turner, Janet Treasure, Hannah Turner, Tracey Wade, Glenn Waller, Roz Shafran

**Affiliations:** ^1^ UCL Great Ormond Street Institute of Child Health University College London London UK; ^2^ Section of Eating Disorders Department of Psychological Medicine Institute of Psychiatry, Psychology & Neuroscience King's College London London UK; ^3^ South London and Maudsley NHS Foundation Trust London UK; ^4^ Department of Child and Adolescent Psychiatry Institute of Psychiatry, Psychology & Neuroscience King's College London London UK; ^5^ Maudsley Centre for Child and Adolescent Eating Disorders South London and Maudsley NHS Foundation Trust London UK; ^6^ Norwich Medical School University of East Anglia Norwich UK; ^7^ Norfolk and Suffolk NHS Foundation Trust Norwich UK; ^8^ Department of Psychiatry Yale School of Medicine New Haven Connecticut USA; ^9^ Beat Norwich UK; ^10^ NHS England Adult Eating Disorders Co‐Chair Parliamentary Health Service Ombudsman's Delivery Group Redditch UK; ^11^ Leeds Institute of Health Sciences School of Medicine University of Leeds Leeds UK; ^12^ Department of Psychiatry Faculty of Medicine University of Geneva Geneva Switzerland; ^13^ Mental Health Services of the Capital Region of Denmark Eating Disorders Research Unit Ballerup Psychiatric Centre Copenhagen Denmark; ^14^ Department of Psychiatry University of Oxford Oxford UK; ^15^ Eating Disorders Service Southern Health NHS Foundation Trust Southampton UK; ^16^ Blackbird Initiative Flinders Research Institute for Mental Health and Wellbeing Flinders University Adelaide South Australia Australia; ^17^ Clinical and Applied Psychology Unit Department of Psychology University of Sheffield Sheffield UK

**Keywords:** access to care, brief therapy, eating disorders, guided self‐help, low intensity, treatment gap

## Abstract

**Objective:**

Eating disorders are associated with significant illness burden and costs, yet access to evidence‐based care is limited. Greater use of programme‐led and focused interventions that are less resource‐intensive might be part of the solution to this demand‐capacity mismatch.

**Method:**

In October 2022, a group of predominantly UK‐based clinical and academic researchers, charity representatives and people with lived experience convened to consider ways to improve access to, and efficacy of, programme‐led and focused interventions for eating disorders in an attempt to bridge the demand‐capacity gap.

**Results:**

Several key recommendations were made across areas of research, policy, and practice. Of particular importance is the view that programme‐led and focused interventions are suitable for a range of different eating disorder presentations across all ages, providing medical and psychiatric risk are closely monitored. The terminology used for these interventions should be carefully considered, so as not to imply that the treatment is suboptimal.

**Conclusions:**

Programme‐led and focused interventions are a viable option to close the demand‐capacity gap for eating disorder treatment and are particularly needed for children and young people. Work is urgently needed across sectors to evaluate and implement such interventions as a clinical and research priority.

## Highlights

- An expert consensus consortium held a workshop to improve access to, and efficacy of, programme‐led and focused psychological interventions for people with eating disorders across the age range.
- Consensus statements and recommendations were produced, including changing terminology and misconception that such interventions are only suitable for specific presentations or mild cases.
- Working together across sectors is needed to close the demand‐capacity gap and integrate programme‐led and focused interventions across care pathways.

## 1 BACKGROUND

### 1.1 Context

Demand for eating disorder services has always exceeded capacity to supply evidence‐based treatments with historically long waiting lists (Hart et al., 2011). The demand‐capacity gap has been further exacerbated by the COVID‐19 pandemic which has led to an increase in eating disorders, particularly in children and young people (CYP) (Katzman, 2021). For example, within the UK, child and adolescent eating disorder services have seen a 60% rise in referrals with urgent referrals more than doubling (NHS England, 2022), and similar increases of referrals have been observed in early intervention services for emerging adults, aged 16–25 (Hyam et al., 2022). It is estimated that 60% of young people aged 17–19 have possible eating problems, an increase from 44.6% in 2017 (NHS Digital, 2022). Consequently, waiting times have increased and waiting time standards are regularly being breached (Eisler et al., 2022; NHS England, 2022). Delays are not without consequences—an extended period without treatment can hamper the chances of recovery, compromise social and occupational attainment, and prolong psychological distress (Austin et al., 2021; Beat, 2017; Flynn et al., 2021). The NHS England Access and Waiting Time Guideline for CYP is based on evidence showing that direct access from primary care to dedicated community eating disorder services and early intervention increases treatment reach, improves continuity of treatment, is valued by patients and families, reduces admissions and reduces costs (National Collaborating Centre for Mental Health, 2015). In line with this, the NHS Mental Health Implementation Plan promises increased investment into new models of integrated primary and community care for adults with severe mental illnesses, including people with eating disorders (NHS England, 2019). To meet the unprecedented demand for treatment quickly and effectively, it is essential to develop and deliver less resource‐intensive interventions that are scientifically supported, accessible and scalable.

Less resource‐intensive interventions encompass a range of interventions such as brief versions of evidence‐based therapies delivered by qualified therapists (i.e., ≤50% therapy contact time of full therapy), and ‘low intensity’ interventions which are supported by self‐help materials or digital platforms, and are typically delivered by non‐specialists, such as paraprofessionals and carers (Shafran et al., 2021). Guided self‐help interventions have been shown to be effective for adults with eating disorders, particularly bulimia nervosa and binge eating disorder (e.g., Fairburn, 2013; Schmidt et al., 2015; Traviss‐Turner et al., 2017). They are also widely used in the treatment of anxiety disorders, depression, and behavioural difficulties in CYP and again, have been shown to be efficacious (Bennett et al., 2019; Thirlwall et al., 2013). Current eating disorder treatment guidelines in the UK recommend guided self‐help for binge‐eating related disorders in adults (National Institute for Health and Care Excellence [NICE], 2017). However, to date, CYP eating disorder services do not routinely use guided self‐help or brief versions of evidence‐based treatment (NICE, 2017). There is also little research comparing guided self‐help for CYP with eating disorders, with two exceptions. The first of these was a randomised controlled trial (RCT) with 85 adolescents comparing cognitive behavioural therapy (CBT) guided self‐care for adolescents with bulimia nervosa and related disorders with family therapy (Schmidt et al., 2007). The outcomes indicated no difference between groups, with the guided self‐care showing greater impact at 6 months and being more cost‐effective. The second was a pilot RCT of 40 adolescents with anorexia nervosa which compared online family‐based guided self‐help to family‐based treatment delivered via videoconferencing (Lock et al., 2021). The results suggested that family‐based guided self‐help was acceptable to families and led to improvements in terms of both weight gain and global eating disorder psychopathology.

Given the urgency of the current situation, it was decided to hold an expert consensus consortium made up of predominantly UK‐based clinical and academic researchers, people with lived experience and charity representatives with expertise in less resource‐intensive interventions in any of the following:

a) Eating disorders in adults (such as 10‐session CBT [CBT‐T], guided self‐help).
b) Eating disorder treatment or prevention in CYP with eating disorders.
c) Common mental health disorders in CYP (such as guided self‐help for CYP with anxiety, depression and behavioural disorders).

The aim of bringing together the expertise in the above areas was to reach a consensus about increasing access to, and efficacy of, less resource‐intensive interventions in eating disorders with the goal of bridging the demand‐capacity gap and to enable more people to get good quality, evidence‐based care. See Supporting Information S1 for details regarding the expert consensus consortium and structure of the meeting.

## 2 DEVELOPING THE GROUNDWORK

During the meeting, several points were made that were broader in scope than the two identified topics and relevant to setting the scene for this discussion.

### 2.1 Terminology used

The meeting was set out to focus on ‘low intensity’ and ‘brief’ interventions for the treatment of eating disorders, as defined in the literature (see Shafran et al., 2021). However, there was agreement that language was important and that the terms ‘low intensity’ and ‘brief’, although widely used, are problematic. They imply that the problem is mild, that patients are receiving a suboptimal form of treatment, that the intervention is ‘low level’ and that others might be receiving ‘more’ treatment. There was consensus that more positive and accurate language to describe low intensity and brief treatments is needed.

It was considered that ‘programme‐led’ was a better alternative to ‘low intensity’ treatment as it indicates that the expertise is in the programme content rather than the therapist or guide, thus enabling wider dissemination. Similarly, it was agreed that ‘focused’ was a better alternative to ‘brief’ as the latter implied there is a longer, fuller version that is not being received. As a guideline, it was agreed that focused treatment is any treatment that requires 50% or less therapist‐time than standard treatment. It was agreed that the emphasis within programme‐led and focused interventions is on full recovery.

Consideration was given to interventions or resources used while awaiting treatment. It was suggested that the term ‘waiting list’ was unhelpful, as this implies a non‐active period of waiting for support. Changing the terminology to ‘preparatory period’ would reflect that this period can be spent reading information, preparing for change, and/or making initial steps towards change using self‐help or supported activities.

Consensus statement 1 *Language is critical. The terminologies ‘programme‐led’ and ‘focused’ are preferable to ‘low intensity’ and ‘brief’ when describing interventions.*

### 2.2 Models of care

Stepped care is a model of healthcare delivery based on the notion that most patients will derive some benefit from a programme‐led or focused intervention and those who do not respond can be ‘stepped up’ to receive a more intensive intervention (Wilson et al., 2000). The key idea underpinning a stepped care approach is that if one treatment does not work for an individual, they can switch to a more intensive, therapist‐led treatment (e.g., NICE guidelines). However, its effective implementation is reliant on more intensive therapist‐led treatments being available to ‘step into’, and the reality is that at present there are insufficient resources to make this seamless transition. As such, alternative models of service delivery are urgently required.

Staged care is a novel model of service delivery that seeks to place a person on a continuum by factors such as severity, duration of symptoms and illness course (e.g., first episode vs. recurrent illness) so as to enable service providers to match treatments to the person's illness trajectory (Iorfino et al., 2021). The main objective of staged care is to ensure that individuals receive the right level of care, rather than selecting a less‐resource intensive intervention, so to optimise treatment and prevention outcomes (Sawrikar et al., 2021).

An alternative option is to front load resources to intervene as early as possible with high level of expertise, and to manage the resources by discharging when a ‘good enough’ level has been achieved and ensure easy return for people who may need additional support further down the line. This would mean using the existing workforce to provide more focused treatments initially which could then free up capacity to see more patients.

It was the view of the group that there is no ‘one size fits all’ model of care that would suit all services but that an important value of programme‐led and focused interventions is that they can be used flexibly across the care pathway in different settings. Some services may choose to have a highly skilled workforce delivering shorter interventions initially, whereas provision of programme‐led interventions as a first step within a stepped care model may be preferable for other services.

### 2.3 Who are these interventions for?

There was agreement that programme‐led and focused interventions should ***not*** be restricted to those of a particular symptom severity (e.g., mild‐moderate), those in particular diagnostic categories (e.g., not underweight) or those at an early stage of their eating disorder. Instead, it was considered that access should be encouraged for a range of people and presentations. It was the group's experience that such interventions can be suitable for people early on in their help‐seeking journey, those who have already received treatment, and those with a long‐standing history of an eating disorder. Feedback from clinicians is that these interventions can consolidate what patients have learnt throughout treatment and that it can help to keep them engaged with services. As suggested by a broad body of evidence, *response to treatment* should be more important in determining suitability for continued intervention than presentation or severity at the start of treatment (Chang et al., 2021; Vall & Wade, 2015). It is, of course, important to take patients' needs and preferences into account when making treatment decisions.

The view was that everybody should be given the chance to access a programme‐led and focused, evidence‐based intervention. This is because some people do not get this opportunity. It was also considered important to determine the initial response to the intervention rather than assuming, a priori, poor response to the treatment offered. A clear algorithm for when to move patients on from the programme‐led intervention is essential, but that presumes that there is additional support readily available. It was agreed that in principle, such interventions should not be offered to patients who present with high risk (e.g., those with rapid weight loss, very low mood, high medical or psychiatric risk, acute suicidality) without mitigation (e.g., medical monitoring). However, the point was made that the risk does not disappear while people are on a waiting list (receiving nothing). It was agreed that whether such interventions can be used with patients who show some risk depends on the service context/environment—if the service can carry and manage risk safely (e.g., inpatient services) then such interventions may be able to be used more readily than in community services. However, intensive medical monitoring would still be required to ensure that critical developments are recognised promptly and accurately, and responded to appropriately. It is paramount that rapid referral to more intensive care is initiated if the patient displays signs of significant deterioration.

An additional consideration is the rich history of programme‐led work involving carers of people with eating disorders who are supporting their loved ones (Treasure et al., 2016). There was consensus that the principles that apply to programme‐led and focused interventions for people with eating disorders apply similarly to the carers and that within a wider system, carers of people with eating disorders should be viewed as a strong resource to help deliver programme‐led and focused interventions. For some CYP, and the many people who never reach services (Hart et al., 2011), it is highly likely that a parent/carer will be delivering and guiding the programme and this needs to be borne in mind.

Consensus statement 2 *Programme‐led and focused interventions are needed to close the demand‐capacity gap. They are:*

- *Not only for first presentations*
- *Not just for early intervention*
- *Suitable for:*
  ‐ *Different presentations of eating disorders in different populations and of different ages (including anorexia nervosa where there is no significant medical or psychiatric risk)*
  ‐ *People caring for loved ones with an eating disorder*

### 2.4 Who could deliver these interventions?

One of the major appeals of programme‐led and focused interventions is that those who support patients to work through these interventions can come from a diversity of backgrounds. This includes any individuals interested in the wellbeing of people, who have undergone relevant training, evidence the necessary competences and are appropriately supervised to support the intervention. Such a workforce would include, but is not limited to non‐specialist psychology graduates, nurses, healthcare support workers, charity workers, dietitians, wellbeing practitioners and social workers, as well as peer support workers, experts by experience, and carers supporting their loved one with an eating disorder (providing that their own support needs were considered and they were linked into wider services as required). As in the treatment of anxiety disorders (e.g., Lawrence et al., 2021), parents/carers are recognised as an important resource and force for change (Eisler, 2005). Empowering parents/carers to deliver or support programme‐led and focused interventions should be encouraged when appropriate for the family's circumstances.

An entirely new practitioner‐based workforce has been funded as part of the UK's Improving Access to Psychological Therapies (IAPT) programme (Clark, 2018; Ludlow et al., 2020), and NHS England have recently launched a Youth Intensity Psychological Practitioner role to support young people with severe mental health needs. These workforces should be tapped into wherever possible.

People with psychology, nursing or dietic background could be trained to support these interventions. They could work in eating disorder services alongside clinicians trained in more complex and intensive psychological interventions. Such a workforce has the advantage of being relatively inexpensive. It is essential that these practitioners are appropriately trained to deliver the intervention and carefully supervised. It may also be the case that for some services, a highly skilled workforce delivering focused interventions may be preferable and it cannot be assumed that what has been a successful model in the treatment of anxiety and depression, as in IAPT, will also be successful in the treatment of eating disorders, as there is significantly less evidence to draw on at present.

Consensus statement 3 *There is a readily available workforce that can be trained to deliver programme‐led and focused interventions for eating disorders. This workforce can provide relatively low‐cost interventions and also free up capacity of specialist clinicians.*

### 2.5 Task sharing

Task sharing is ‘a process whereby specific tasks are moved, where appropriate, to health workers with shorter training and fewer qualifications’ (World Health Organization, 2007, p. 7). Task sharing is considered a promising approach to disseminate evidence‐based mental health support to areas where a skilled workforce may be lacking (Patel, 2022). In the field of eating disorders, parents of CYP have been tasked with delivering supervision and eating support within family‐based treatment and this form of treatment has been shown to provide specific benefits over other approaches (Monteleone et al., 2022). Work is in progress to deliver this treatment in alternative ways to the standard face‐to‐face form (Hambleton et al., 2020). However, feedback from patients and carers is that they would like to be given help to provide psychological support rather than solely a weight‐focused approach (Mitrofan et al., 2019), which is not effective in all cases (Wufong et al., 2019).

In the Maudsley model for anorexia nervosa treatment in adults (MANTRA), the patient workbook has a section for supporters which includes information on how to provide both psychological and physical support. Moreover, Supporting Information S1 in the form of books (Langley et al., 2018; Macdonald, 2021; Treasure et al., 2016) and websites have been co‐developed with people with lived experience to provide more in‐depth information and skills sharing for the network of various forms of social and medical support that are needed for this complex condition. Task sharing with parents has been found to improve outcomes in both the adolescent and adult setting (Hibbs et al., 2015; Hodsoll et al., 2017; Magill et al., 2016). Task sharing with peer mentors (individuals in the early stage of clinical training) can reduce anxiety and increase alliance when they are used to augment the early phase of engagement in outpatient treatment (Cardi et al., 2020). Interestingly, research suggests peer mentors with lived experience of an eating disorder develop a better working alliance with patients, suggesting that task sharing may need to be widened in this way (Albano et al., 2021, 2022). People with lived experience of anorexia nervosa may write about their own recovery story to provide hope and information (Bryant, 2021). Alignment of this perspective in combination with evidence‐based models and targeted treatments has the potential to lead to improved collaborative, task sharing treatments for anorexia nervosa.

### 2.6 Focused training

Focused training is important in the context of programme‐led and focused interventions. Fundamentally, the message should be clearly communicated that training provides the skills and competencies for the individual to act as a ‘guide’ and that the role is to support the patient to use the materials, rather than being a ‘therapist’. There is some research on what makes a good guide (e.g., listening, reinforcement, instilling hope; Traviss et al., 2013), and that patient and carer feedback is essential in training guides in those skills (Tam & Ronan, 2017). It was agreed that Health Education England (HEE)'s focus on core competences rather than job titles was helpful (see UCL competence frameworks for the delivery of guided self-help for eating disorders). Knowledge of the intervention is an important element of competence which should be evaluated (e.g., Jenkins, 2020). The duration of training and content should consider the knowledge that people already have and training should be tailored accordingly. For example, dietitians working in eating disorders already have a good understanding of different eating disorders, whereas psychology graduates may be less aware of the range of presentations. It was recommended that training duration needed to be realistic in terms of time away from clinical practice that would be permitted in busy services. Services are encouraged to develop their own internal training resources. The training can be added to the curricula for staff who already do this work in other areas of mental health (e.g., anxiety and depression), such as Psychological Wellbeing Practitioners (PWPs), Children's Wellbeing Practitioners (CWPs), Mental Health Wellbeing Practitioners (MHWPs) and Educational Mental Health Practitioners (EMHPs). It was also considered important that such training should include the use of outcome monitoring and feedback, alongside how to individualise treatment to individual or patient group needs within a standardised programme‐led protocol.

Staff turnover for this workforce is high (HEE, 2022) and the training needs to be sustainable. Recording trainings, using a ‘train the trainer’ model, frequently asked questions documents to cover ‘what ifs?’ and an adherence checklist (e.g., Bailey‐Straebler et al., 2022) outlining what should be covered, were all considered helpful when considering the sustainability of training the workforce. Such a checklist can be reviewed in supervision (including self‐supervision) to ensure both fidelity to the programme protocol and to prevent drift from being a ‘guide’ to being a ‘therapist’.

Consensus statement 4 *A training and competency development model for the delivery of programme‐led and focused interventions is required, and could be incorporated into existing training programmes for other mental health disorders.*

### 2.7 Focused supervision

Appropriate supervision is particularly important given that the workforce is relatively junior. However, if it is too extensive and competence‐based (e.g., co‐observation, listening to entire sessions), then it becomes too similar to the type of supervision seen in the training of specialist clinicians (Frank et al., 2020). Although group supervision is often provided, the capacity for supervision is itself limited and can create a further barrier to access. Organisations such as the British Association of Behavioural and Cognitive Psychotherapies (BABCP), British Psychological Society (BPS) and British Association for Counselling and Psychotherapy (BACP) provide guidelines regarding ratios of supervisors/supervisees and appropriate supervisory input. Workforce planning needs to consider both the number of workers available to recruit, along with how many senior people can deliver training and supervision. Supervision should make clear the role and expectations of the supporter in programme‐led and focused interventions. Such supervision should always be data‐informed and session‐by‐session outcome measurement is central.

It may be helpful to draw a distinction between case management supervision and clinical supervision, as is the case in the IAPT programme, where the former involves regular reviews of entire caseloads and the latter focuses on patient progress, therapeutic technique and treatment fidelity (Curry & French, 2022).

Consensus statement 5 *Individuals supporting the delivery of programme‐led and focused interventions must be offered high quality supervision and consultation.*

### 2.8 Patient reported outcome measures

One of the key developments in psychological treatment is the recognition of the value of session‐by‐session patient reported outcome measures (PROMs) in improving treatment outcome and as an essential part of clinical decision‐making as well as supervision (e.g., Lutz et al., 2022). Such outcome‐informed psychological therapy is fundamental in facilitating a programme‐led approach as it allows regular monitoring of progress and consideration of different options when there is insufficient initial progress. It may be the case that there is a change from a programme‐led to a therapist‐led approach, or that there is increased focus on a particular aspect of the intervention. This outcome‐driven approach allows a personalisation of the programme, while maintaining fidelity to the protocol and also to the role of the guide rather than therapist. To mitigate the risk that this will not succeed to a sufficient extent in practice, it is suggested that (a) such processes are as automated as possible; and (b) clinicians are trained in the importance of PROMs as a technique to improve outcome (e.g., Delgadillo et al., 2018), as well as its importance ethically with regard to clinical decision making. Patients' progress on these measures should be a routine part of clinical supervision to facilitate monitoring quality and progress. However, it is important to bear in mind that critical signs in patients may not be reflected in self‐report questionnaires and so such measures should not be solely relied upon when making clinical decisions.

In addition to actively monitoring progress and outcomes for patient benefit, scrutinising such data at the service and practitioner level was considered beneficial to facilitate shared data and learning. Service users being able to look up the effectiveness of the treatment centre in the same way as with other areas of health (e.g., surgical procedures; https://www.nhs.uk/mynhs/specialties) was considered to be empowering and something to be considered in the field of eating disorders.

Consensus statement 6 *Session‐by‐session outcome monitoring is an essential part of clinical decision making, supervision, transparency and accountability for programme‐led and focused interventions.*

### 2.9 Starting and ending well

It can often be challenging to engage people in the treatment of eating disorders (Innes et al., 2017). This is particularly the case when there is ambivalence about treatment and recovery. It may be the case, however, that guided self‐help is precisely useful for those who do not seek treatment due to their ambivalence and fears. It was considered essential for any programme‐led and focused intervention, as with other psychological interventions, to consider the right time for beginning treatment to maximise the chance of success given that starting well is predictive of later outcome (Vall & Wade, 2015). It was acknowledged that not everyone needs to be highly motivated at the start of treatment. Rather, the focus should be on starting well—checking that the patient sees the intervention as suitable and feels relatively confident that it can work for them, and work for them now. It is important to work through any potential barriers to change, and to encourage the patient to experiment with initial early changes given that early change is critical to treatment outcome. It was agreed that patients should be given accurate and transparent information about the intervention in order to set realistic expectations. It needs to be made clear that whilst such interventions may not solve all of their problems immediately, adhering to the programme will give them the best chance of full recovery.

Consensus statement 7 *Early change is critical to treatment success, so greater efforts need to be made early on to help the patient to navigate challenges and to build the self‐efficacy to experiment with initial early change.*

Although there are very little data available, we know the recovery rates with programme‐led and focused interventions is around 50% (e.g., Cachelin et al., 2019; Carter et al., 2020; Peterson et al., 2020). For people who do not respond, as well as those who have made a full recovery or who are unable to complete the intervention, there was agreement that it is important that the treatment comes to a positive end, whenever that is. Understanding the person's disengagement and drop‐out, as well as sending a therapeutic letter highlighting what they did well, encouraging them to not be disheartened and detailing a range of personalised options for alternative support, were suggested methods to maximise the chance of ending well. It is also essential that the individual is given the programme materials beyond the end of treatment to maintain any changes made.

Consensus statement 8 *Regardless of when treatment comes to an end, it is important that it ends positively. The focus should be on what the patient did well throughout the treatment and suggestions for alternative support if needed.*

### 2.10 Cost and policy

It was agreed that what is needed is a ‘multi‐pronged’ approach to the use of programme‐led and focused interventions that are cost effective, consistent and equitable across the country. The cost savings of a stepped care approach should be compared with traditional services and other service models such as provision of briefer, therapist‐led interventions ‘upfront’. The onus is on researchers to demonstrate that programme‐led and focused interventions work well, and result in significant clinical, social, educational and economic benefits. Routinely collected PROMs data can form part of this evaluation and can be used to showcase whether these interventions are effective, and for whom.

Consensus statement 9 Studies to establish the cost‐effectiveness of programme‐led and focused interventions across different models of care are needed to effect policy change.

## 3 INCREASING ACCESS TO PROGRAMME‐LED AND FOCUSED INTERVENTIONS

### 3.1 Assessment

Assessment was considered a major barrier to accessing programme‐led interventions. Access to assessment is clearly a precursor to accessing intervention within services. Online self‐referral or carer‐referral and computerised screening were viewed as useful tools in expediting the process of receiving an intervention. Self‐referral was considered empowering for individuals and their families. Although being linked to primary care would be a requirement, referral via such a practitioner should not be a prerequisite to accessing support. Although some thought around the governance and safety of self‐referral/carer‐referral is necessary.

Consensus statement 10 *A self‐referral/carer‐referral route to programme‐led and focused interventions, which bypasses primary care, can open up pathways to care.*

### 3.2 Scalability versus guidance

There is a balance between scalability and keeping individuals engaged in treatment. Self‐help interventions with no guidance can be offered at scale, however outcomes and retention are better when there is a guide (Beintner et al., 2014; Musiat et al., 2022). An initial face‐to‐face meeting was identified as a possible solution, even if guidance takes place online or remotely. Guidance on demand has been shown to be as effective as scheduled guidance (e.g., Berger et al., 2011), and this may provide the right balance between scalability and human resource. It was considered important to understand the pros and cons of the different forms of guidance which range from brief face‐to‐face sessions, scheduled telephone calls or emails, to ‘chatbots’. It was agreed that the advantages and disadvantages of the different forms of guidance are important to evaluate.

Consensus statement 11 *We need more information on how best to provide guidance in programme‐led and focused interventions, including how, when and in what form.*

### 3.3 Format of programme delivery

Many of the tried‐and‐tested programme‐led approaches for eating disorders are in printed format, whereas newer approaches are often delivered digitally or online. Digital programme‐led interventions offer the potential to reach a larger population of individuals, as well as those who are currently underserved. However, it cannot be assumed that digital formats are preferred by patients or are cost effective (Hollis et al., 2017; Sweeney et al., 2019). Furthermore, at present, digital programmes almost exclusively remain in the domain of research and few are publicly available (see Bear et al., 2022).

Consideration needs to be given to the sustainability and implementation of any novel programmes, in digital or printed format, in addition to their clinical and cost‐effectiveness. In the future, digital solutions may be best used as part of the therapeutic toolkit of evidence‐based treatments, in addition to printed programmes to allow for patient, clinician and service preferences. Efforts are required to adapt the language of programme‐led and focused interventions to meet the needs of diverse populations, and their reach should be evaluated.

Consensus statement 12 *It is preferable to ask people how they would most like to receive intervention materials and for services to be sufficiently flexible to meet such preferences.*

### 3.4 Spreading the word

People often hear about eating disorder treatments on social media and, as such, social media needs to be utilised to let people know about the availability and utility of programme‐led and focused interventions. Evidence‐based, targeted messaging was suggested to encourage people to get to the point when they are thinking about treatment and could help to overcome some common barriers to help‐seeking, such as down‐playing of illness severity and less perceived ability of others to help (Radunz et al., 2022; Webb & Schmidt, 2021). Involving people with lived experience is critical at every step, especially when it comes to spreading the word on social media. CYP in particular can be motivated by influencers talking about recovery on TikTok and other platforms (Herrick et al., 2021), and sharing such positive experiences is an under‐used yet potentially powerful means of improving knowledge and subsequently access to such treatment (Au & Cosh, 2022; Niederkrotenthaler et al., 2021).

We know that stigma related to mental illness and mental health services can have a deterrent effect on help‐seeking (Schnyder et al., 2017). There are plenty of lessons to be learnt from programmes designed to combat mental‐health‐related stigma and promote help‐seeking (Thornicroft et al., 2022).

Consensus statement 13 *We need to explore innovative ways to bring people's attention to the existence of services, as well as increase their willingness to engage with services.*

### 3.5 Points of access

**FIGURE 1:**
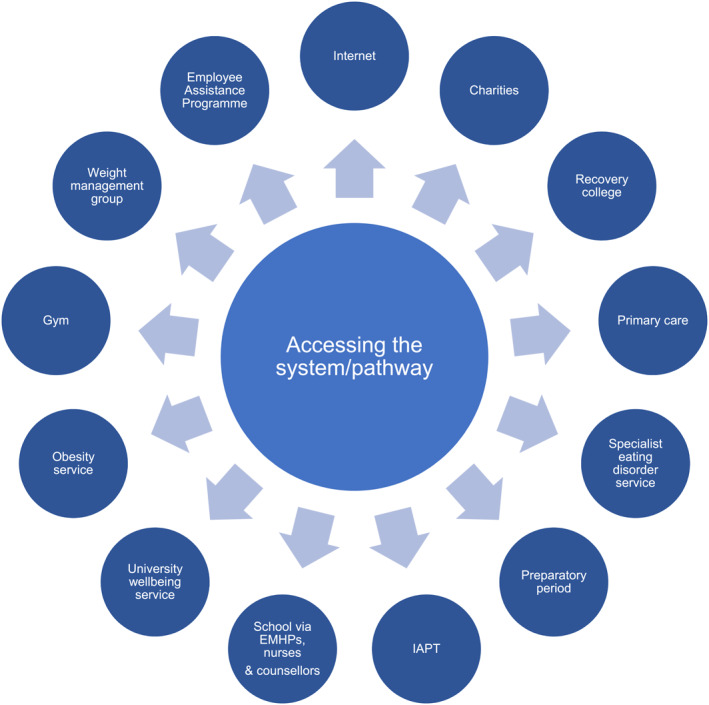
Potential avenues to access programme‐led and focused interventions. EMHP, Educational Mental Health Practitioner; IAPT, Improving Access to Psychological Therapies.

There was consensus that there should be a broad range of places through which the intervention can be obtained. It follows that the programme‐led and focused intervention will, at times, be embedded and sit within traditional services but at other times will not. Such flexibility is needed to ensure that the maximum number of people possible benefit from the intervention. Where in the system the help is accessed may depend on who is accessing it. If it is someone who perceives stigma attached to seeking help for an eating disorder, then the Internet may be the first port of call. Given the strong feature that down‐playing of illness severity has in eating disorders, it was acknowledged that the help‐seeking was often led by the carer or supporter. If the programme‐led intervention is for a young person and their carers are the ones motivated to engage and are acting as the guide, then primary care services may be a key point of access. Schools may also be an important route into care for CYP. Learning from successful models, such as IAPT, was suggested as a helpful way forward to facilitate help‐seeking at any of these points. It was acknowledged that to truly increase access, such interventions should be available both within services as part of a broader offering, but also beyond services to reach the many people currently underserved by existing service provision (Hart et al., 2011; Striegel Weissman & Rosselli, 2017). See Figure 1 for some proposed avenues to access programme‐led and focused interventions.

Consensus statement 14 Programme‐led and focused interventions should be accessed via multiple routes. They should be seen as one component of a broader care offering that are embedded within (e.g., primary care and specialist eating disorder services) and extend beyond services (e.g., schools).

Taking into account all of the above, increasing access to programme‐led and focused interventions is a priority to address unmet treatment needs. However, it is equally important that once individuals are at the point of receiving such interventions, these interventions have a sound evidence‐base and are optimally effective. We must therefore consider ways to maximise the potential of programme‐led and focused interventions to improve therapeutic outcomes.

## 4 IMPROVING OUTCOMES WITH PROGRAMME‐LED AND FOCUSED INTERVENTIONS

In addition to the general points made above, such as the benefits of PROMs, timing, training and supervision, additional areas were considered important in improving outcomes with programme‐led and focused interventions for people with eating disorders.

### 4.1 Improving adherence

Poor patient adherence to self‐help materials is widely acknowledged as a significant factor in treatment discontinuity and poor treatment outcomes (Puls et al., 2020), representing a major challenge in programme‐led and focused interventions. Close monitoring of adherence throughout the programme can help to identify those who need additional support so that the guide can implement techniques to enhance engagement and promote adherence. Involving carers in treatment, especially for CYP, is one idea. Further attention to strategies that facilitate adherence will help realise the potential of these interventions.

Consensus statement 15 *Adherence to core treatment elements is important to maintain treatment efficacy. Using strategies that facilitate engagement and adherence have the potential to improve treatment outcomes and reduce drop‐out, such as involving carers.*

### 4.2 Practice‐based evidence

It is difficult to obtain funding to test new ideas in expensive, large‐scale randomised controlled trials. Such gold‐standard research methods are needed but can also be slow. Innovations to improve treatment outcomes from clinical observations and lived experience should be valued and harnessed. Focusing on the implementation and adaption of approaches already tested requires further consideration.

Consensus statement 16 *Adopting a systematic and practice‐based approach (i.e., co‐production, utilising clinical expertise and gathering evidence during practice) and pragmatic implementation approach may help to enhance outcomes in the real world.*

### 4.3 Tailoring treatment

There was recognition that treatment needs to be tailored towards specific groups. These may include, for example, neurodiverse populations, those with specific difficulties in regulating emotions, or living with physical health conditions where dietary needs and eating patterns may be inherently different, and those at high medical or psychiatric risks. Groups, such as those with Avoidant/Restrictive Food Intake Disorder, are currently underserved and neglected in terms of treatment research. The programme should have built in flexibilities to be able to modify the programme for specific groups, as opposed to individual guides changing the programme more idiosyncratically to suit individuals. This could be achieved with a modular approach in which optional modules can be added to the core intervention programme as necessary. Tailoring treatment to those with atypical presentations was considered likely to reap benefits in terms of improved outcomes. However, it is important that modifications for specific groups are carefully evaluated. Greater use of adaptive trial designs (Nahum‐Shani et al., 2012) should be considered in intervention studies, where the type and/or dosage of the intervention offered is adjusted over time depending on the individual's response.

Consensus statement 17 *A personalised approach that considers variations in patient characteristics and preferences may be optimal for understanding and treating specific groups, including neurodiverse populations, individuals with emotion dysregulation and those at high psychiatric or medical risk.*

### 4.4 Treatment matching

The matching of programme‐led and focused interventions to particular patient characteristics, needs and preferences may help to bolster their effectiveness. Treatment matching has long been a goal within the field of eating disorders (Waller, 2016), yet at present we still do not have sufficient information to allow a confident matching of treatment with individual need. Can we find something that will predict whether someone will do well with a particular treatment? If not, can we be responsive early enough to modify or end treatment, according to the needs of patients based on the data? Can we pool our research data to allow such questions to be answered with confidence? There are many questions concerning treatment matching but work in other areas of mental health, such as depression, have proven fruitful (e.g., the development of the Personalised Advantage Index; DeRubeis et al., 2014). Such an approach can serve as a model for eating disorders.

Consensus statement 18 *Learning from treatment matching in depression can serve as a model for eating disorders.*

### 4.5 Treatment innovations and development

Transdiagnostic treatment protocols proceed from the notion that disorders share common processes and so the same underlying treatment principles can be applied across disorders (Dalgleish et al., 2020). Transdiagnostic treatments (such as enhanced CBT; Fairburn et al., 2003) where some procedures are considered universal, with relevance to all patients, and some procedures and modules are used selectively as needed, have proven highly successful (e.g., Dahlenburg et al., 2019). Fully modular approaches, such as Modular Approach to Therapy for Children with Anxiety, Depression, Trauma or Conduct Problems (MATCH‐ADTC; Chorpita & Weisz, 2009) that comprise sets of therapy modules that can operate independently and flexibly so treatment can be tailored to the needs of each patient also have the potential to improve outcomes.

To facilitate innovation in the field of programme‐led and focused interventions, it can be helpful to have a framework to guide the development and optimisation of such treatments. Example frameworks include the Multiphase Optimization Strategy (MOST; Collins et al., 2005) and the Medical Research Council's (MRC) updated framework for developing and evaluating complex interventions (Skivington et al., 2021).

Consensus statement 19 Frameworks for treatment development, including modular treatments, and optimising interventions should be used to facilitate treatment innovations.

### 4.6 Mechanisms research

There was consensus that an enhanced understanding of the processes of behaviour change in existing psychological treatments may have the potential to make programme‐led and focused interventions more expeditious and effective. Research into mechanisms of action can help to streamline treatment strategies that directly target agents of change and remove any strategies that do not contribute to treatment success. It may also identify crucial moderators of treatment outcome that can improve precision in matching and tailoring programme‐led and focused interventions to the needs of each individual (Holmes et al., 2018). With regard to prevention, it may be helpful to take a transdiagnostic approach towards possible interventions. However, it is important to note that mechanisms research can be a slow and difficult process. Given the urgency of the situation, we may need to work pragmatically to meet people's needs in the immediate term, although this should not undermine further efforts to understand the components of behaviour change and mechanisms of action.

Consensus statement 20 If we can better understand the components of behaviour change and mechanisms of action, we can use this information to improve the programme‐led and focused interventions that already exist, develop new ones and tailor the programmes to the needs of the individual.

## 5 SUMMARY OF RECOMMENDATIONS

The consensus statements have been collated in Table 1.

**TABLE 1:** List of consensus statements.

Key take‐home messages
*Consensus statement 1*: Language is critical. The terminologies ‘programme‐led’ and ‘focused’ are preferable to ‘low intensity’ and ‘brief’ when describing interventions
*Consensus statement 2*: Programme‐led and focused interventions are needed to close the demand‐capacity gap. They are: Not only for first presentations Not just for early intervention Suitable for: ‐ Different presentations of eating disorders in different populations and of different ages (including anorexia nervosa where there is no significant medical or psychiatric risk) ‐ People caring for loved ones with an eating disorder
*Consensus statement 9*: Studies to establish the cost‐effectiveness of programme‐led and focused interventions across different models of care are needed to effect policy change
Workforce, training and supervision
*Consensus statement 3*: There is a readily available workforce that can be trained to deliver programme‐led and focused interventions for eating disorders. This workforce can provide relatively low‐cost interventions and also free up capacity of specialist clinicians
*Consensus statement 4*: A training and competency development model for the delivery of programme‐led and focused interventions is required, and could be incorporated into existing training programmes for other mental health disorders
*Consensus statement 5*: Individuals supporting the delivery of programme‐led and focused interventions must be offered high quality supervision and consultation
Optimising guidance and support
*Consensus statement 6*: Session‐by‐session outcome monitoring is an essential part of clinical decision making, supervision, transparency and accountability for programme‐led and focused interventions
*Consensus statement 7*: Early change is critical to treatment success, so greater efforts need to be made early on to help the patient to navigate challenges and to build the self‐efficacy to experiment with initial early change
*Consensus statement 8*: Regardless of when treatment comes to an end, it is important that it ends positively. The focus should be on what the patient did well throughout the treatment and suggestions for alternative support if needed
*Consensus statement 11*: We need more information on how best to provide guidance in programme‐led and focused interventions, including how, when and in what form
*Consensus statement 15*: Adherence to core treatment elements is important to maintain treatment efficacy. Using strategies that facilitate engagement and adherence have the potential to improve treatment outcomes and reduce drop‐out, such as involving carers
Reaching those in need
*Consensus statement 10*: A self‐referral/carer‐referral route to programme‐led and focused interventions, which bypasses primary care, can open up pathways to care
*Consensus statement 13*: We need to explore innovative ways to bring people's attention to the existence of services, as well as increase their willingness to engage with services
*Consensus statement 14*: Programme‐led and focused interventions should be accessed via multiple routes. They should be seen as one component of a broader care offering that are embedded within (e.g., primary care and specialist eating disorder services) and extend beyond services (e.g., schools)
Patient centred‐care
*Consensus statement 12*: It is preferable to ask people how they would most like to receive intervention materials and for services to be sufficiently flexible to meet such preferences
*Consensus statement 17*: A personalised approach that considers variations in patient characteristics and preferences may be optimal for understanding and treating specific groups, including neurodiverse populations, individuals with emotion dysregulation and those at high psychiatric or medical risk
Treatment innovations
*Consensus statement 16*: Adopting a systematic and practice‐based approach (i.e., co‐production, utilising clinical expertise and gathering evidence during practice) and pragmatic implementation approach may help to enhance outcomes in the real world
*Consensus statement 18*: Learning from treatment matching in depression can serve as a model for eating disorders
*Consensus statement 19*: Frameworks for treatment development, including modular treatments, and optimising interventions should be used to facilitate treatment innovations
*Consensus statement 20*: If we can better understand the components of behaviour change and mechanisms of action, we can use this information to improve the programme‐led and focused interventions that already exist, develop new ones and tailor the programmes to the needs of the individual

In addition to the consensus statements, the following recommendations arose from the meeting.

### 5.1 Research

*We highlighted that research needs to address the following points*:

1. Before treatment matching and big data analysis can take place, there needs to be agreement on outcome measures. A core minimum dataset should be agreed and adopted by researchers.
2. It is important to consider the architecture of what makes a good intervention, as well as the content itself
  - Diversity of different approaches are encouraged.
  - Need to determine what good guidance looks like to maximise treatment efficiency without compromising effectiveness.
  - Need to identify variables associated with adherence and drop‐out and test different strategies to promote adherence.
  - Need to explore potential harms that may arise from these interventions and offer ideas on how best to monitor and mitigate these harms.
3. The value of programme‐led and focused interventions for CYP and people with anorexia nervosa and other non‐binge/purge eating disorders is at present uncertain. Further research investigating the use of these interventions for these currently under‐served populations is necessary.
4. We need to establish the reach, as well as long‐term clinical and cost‐effectiveness, of programme‐led and focused interventions, and include people with lived experience to ensure findings are meaningful.

### 5.2 Services

Generally, services are risk‐averse, which may lead to conservative behaviours such as not interacting at all with people on their wait‐list (to avoid clinical responsibility if something goes wrong while they wait for treatment), or becoming rigid in applying all treatment components to all people referred to a service (to show that everything was done in the face of any potential adverse events). One way to mitigate this is for primary care physicians to retain responsibility for the individual throughout the programme while they remain on the waiting list.

Serious consideration also needs to be given, by those in leadership, to the balance between committing resources to early intervention as well as managing crisis work. Such a move is economically defensible, as well as having a potential to reduce future waiting lists (Allen et al., in press).

We recommend the need to embed PROMs as part of routine clinical practice, including session‐by‐session measures. When flexibility and responsivity is linked with robust evaluation, it has the potential to free up resources to more clients and patients, and to decrease staff burnout (Eshkevari et al., 2022).

### 5.3 Practitioners

We encourage practitioners to use programme‐led and focused interventions in a way that best fits within their practice, and their health system and context.

This includes a consideration of diversifying the clinical workforce and encouraging task shifting from highly qualified clinicians to those who bring core skills and can be supervised in guiding use of programme‐led interventions.

### 5.4 Policy makers

Eating disorders are associated with substantial financial costs, mostly from inpatient treatment, which are borne by patients, carers and wider society. These costs include loss of earnings for patients and carers, as well as productivity losses and costs to the healthcare system (Beat, 2015). Programme‐led and brief interventions are lower cost both in the materials used, and in the training/expertise necessary to be a guide or supporter. Any funds allocated towards these interventions are likely to be offset by reduced spending elsewhere. For example, these interventions could assist in avoiding the need for people to access more costly care types, such as inpatient settings. It is, of course, necessary that the potential erosion in terms of quality of care in favour of economic savings is carefully considered.

### 5.5 Limitations

These consensus statements and recommendations are not without limitations. The attendees were selected, and predominantly UK‐based. Future endeavours may benefit from a wider group of unselected stakeholders across different health care systems. Although experienced clinicians and researchers, the statements are not co‐produced and lived experience input was relatively limited. Future work in this area should go through established co‐production processes, such as the James Lind Alliance, to ensure the voices of people with lived experience are heard and incorporated into the development and evaluation of programme‐led and focused interventions.

## 6 CONCLUDING COMMENTS

The meeting was widely considered productive with a range of consensus statements produced to close the demand‐capacity gap and with a focus on CYP. It is clear that clinicians and researchers are working hard to develop and evaluate programme‐led and focused interventions for currently under‐served populations. The ambition is to reconvene on an annual basis to review progress and impact.

## CONFLICT OF INTEREST STATEMENT

The authors declare no conflict of interest.

## Supporting information

Supporting Information S1

## Data Availability

Data sharing not applicable to this article as no datasets were generated or analysed during the current study.
