# Supplementary material for: Improving programme‐led and focused interventions for eating disorders: An experts' consensus statement—A UK perspective
Source: Eur Eat Disord Rev. 2023 May 22;31(5):577–95. doi: 10.1002/erv.2981 (PMC10947440; doi:10.1002/erv.2981)
Supplement: Supplementary file 1 — Supporting Information S1 [file ERV-31-577-s001.docx]

**Expert consensus consortium**

Twenty-four people were invited to the meeting and in total, 20 people were able to attend. The cohort included clinical psychologists, psychiatrists, counsellors, mental health nurses, academic researchers, PhD students, people with lived experience, and representatives from Beat, the UK eating disorders charity.

While the meeting was held in the UK and was predominantly a UK-based group, it was felt that the general principles outlined in this paper will apply to other countries, although they may have different specific solutions under this broad call to action.

This paper summarises the content of the meeting, current thinking in the area, and concludes with key recommendations for clinical practice and research to help meet the demand for scalable, effective interventions for people with eating disorders. Although there was a focus on CYP, the discussion was not restricted to young people but also included underserved groups such as those with Avoidant/Restrictive Food Intake Disorder (ARFID) and atypical anorexia nervosa, those who require weight management, those who have chronic physical health conditions such as diabetes, and adults in middle age. The dichotomy of CYP versus adults was considered to be unhelpful, as the transition phase from CYP eating disorder services to adult services is challenging (Wade, 2022; Herpertz-Dahlmann & Schmidt, 2022) and it was felt that approaches available for adults could be useful to inform care for CYP. There are precedents for this within eating disorders that have cut across traditional age boundaries, such as the work with supporters of people with eating disorders (e.g., Harrison et al., 2022), family-based interventions (e.g., Eisler et al., 2016) and CBT (e.g., Pretorius et al., 2009; Dalle Grave et al., 2021). There was agreement that individuals across all ages are in need of less-resource intensive interventions.

The hope is that this paper encourages practitioners and academics in the field of eating disorders to consider using evidence-informed, less resource-intensive interventions, to think creatively about using a different workforce to deliver such interventions, and to evaluate such services to increase confidence in their fitness for purpose.

**Meeting structure**

Each participant circulated a key paper in the field of ‘low intensity’ and ‘brief’ interventions, either from their own group or someone else’s, in advance of the meeting. They were then asked to give a brief 10-minute overview of their work in the area and ideas for improving access to, and efficacy of, ‘low intensity’ and ‘brief’ interventions.

There was a total of 11 presentations focusing on increasing access to, and efficacy of, ‘low intensity’ and ‘brief’ interventions from different perspectives. The experts then divided into three groups to discuss increasing access to ‘low intensity’ and ‘brief’ treatments for eating disorders. The groups reconvened and pooled their ideas. This was later repeated with the topic of discussion being improving the efficacy of such interventions. The day ended with a session summarising and synthesising the discussion.
